# Genetic and environmental factors affecting the expression of α-gliadin canonical epitopes involved in celiac disease in a wide collection of spelt (*Triticum aestivum* ssp. *spelta*) cultivars and landraces

**DOI:** 10.1186/s12870-018-1487-y

**Published:** 2018-11-01

**Authors:** Benjamin Dubois, Pierre Bertin, Louis Hautier, Yordan Muhovski, Emmanuelle Escarnot, Dominique Mingeot

**Affiliations:** 1 Unit of Bioengineering, Department of Life Sciences, Walloon Agricultural Research Center, Gembloux, Belgium; 2 Earth and Life Institute-Agronomy, Catholic University of Louvain, Louvain-la-Neuve, Belgium; 3 Unit of Plant protection and ecotoxicology, Department of Life Sciences, Walloon Agricultural Research Center, Gembloux, Belgium; 4 Unit of Breeding and biodiversity, Department of Life Sciences, Walloon Agricultural Research Center, Gembloux, Belgium

**Keywords:** Spelt, Wheat, α-Gliadin, Celiac disease, TaqMan probe, N fertilization, Epitope, Gluten

## Abstract

**Background:**

Celiac disease (CD) is an autoimmune disorder affecting genetically predisposed individuals whose dietary gluten proteins trigger an inflammatory reaction in the small intestine. Gluten is found in the seeds of cereals like bread wheat (*Triticum aestivum* ssp. *aestivum*) and spelt (*Triticum aestivum* ssp. *spelta*). The development of new varieties lacking immunogenic peptides is one of the strategies currently investigated to address the CD problem. Among gluten proteins, α-gliadins display the strongest immunogenicity with four main T-cell stimulatory epitopes. The objective of this work was to study the expression of α-gliadin epitopes related to CD in a wide collection of 121 spelt accessions (landraces and varieties, spring and winter accessions) from different provenances, and to analyze the correlation between the presence of epitope sequences in gDNA and their expression (cDNA). The effect of environmental factors (harvest year and N fertilization) on the epitope expression was also investigated.

**Results:**

TaqMan probes targeting the canonical form of the epitopes were used to evaluate the epitope expression levels. Significant variations in the amount of epitope transcripts were identified between accessions and according to the provenances. Spring accessions showed a significantly higher immunogenicity than winter ones and no influence of spelt breeding on the epitope expression levels could be assessed when comparing landraces and varieties from Northwestern Europe. No correlation was observed between quantitative PCR results obtained from cDNA and gDNA for 45 accessions tested, stressing the need to use markers focusing on epitope transcripts rather than on genomic sequences. A relative stability of the amount of epitopes expressed by a same accession across four harvest years was detected. The fertilization strategy, evaluated through seven N fertilization modalities applied to two commercial spelt varieties, did not influence the epitope expression of the first variety, whereas it had a slight effect for the second one.

**Conclusions:**

The results obtained in this work showed that the CD-related epitope expression greatly fluctuated among the spelt accessions studied. This expression was not correlated to the epitope genomic occurrence and environmental factors had almost no influence on the amount of epitope transcripts.

**Electronic supplementary material:**

The online version of this article (10.1186/s12870-018-1487-y) contains supplementary material, which is available to authorized users.

## Background

Celiac disease (CD) is an immune disorder of the upper small intestine triggered by the ingestion of gluten. The inflammatory response of the immune system to the presence of dietary gluten leads to a flattening of the intestinal mucosa, resulting in the malabsorption of nutrients. This pathology affects genetically predisposed individuals. While its occurrence has risen during last decades to reach ~ 1% of the human population, most patients affected by CD are still undiagnosed [1, 2]. Gluten is composed of storage proteins from wheat (*Triticum aestivum* L. ssp. *aestivum*), spelt [*Triticum aestivum* ssp. *spelta* (L.) Thell.], barley (*Hordeum vulgare* L.) and rye (*Secale cereale* L.). Oat (*Avena sativa* L.) also displays a small fraction of prolamin storage proteins, called avenins, which do not contain any CD-related epitopes [3]. The oat immunogenicity has been the subject of many discussions, but it seems that, when gluten peptides are found in oat products, they come from contaminations with wheat, barley or rye in the production chain, rather than from oat itself. Long-term food studies have confirmed the safety of oats for CD patients and the putative health benefit of oat products in a gluten-free diet [3–5]. The daily intake of gluten should be kept under 50 mg for CD patients [6] and they must thus follow a strict life-long gluten-free diet to avoid intra- and extra-intestinal symptoms such as diarrhea, bowel pain, fatigue, weight loss, anemia, osteoporosis, headaches and growth retardation [7–9].

Gluten proteins are usually classified into soluble monomeric gliadins and insoluble polymeric glutenins according to their alcohol solubility. Gliadins confer viscosity to dough and are divided into three structural groups according to their electrophoretic mobility: α/β-, γ- and ω-gliadins. Glutenins are responsible for dough elasticity and classified into high- and low-molecular-weight glutenin subunits (HMW- and LMW-GS) [10]. Gluten proteins display a high content of proline and glutamine amino acids, which make them partially resistant to gastrointestinal digestion [11].

The α-gliadins are recognized as the most toxic group of gluten proteins affecting CD patients since they trigger the strongest T-cell activation [12–15]. The α-gliadins are encoded by a multigene family. Indeed, the Gli-2 loci, where the α-gliadin genes are located, include a high number of α-gliadin gene copies. However, it has been shown that a high proportion of these genes are pseudogenes as they display a premature stop codon in their reading frame [16, 17]. Recent researches found different amounts of active α-gliadin genes according to the accession studied and the methodology used. Kawaura et al. [18] sequenced bacterial artificial chromosome (BAC) clones covering about 200 kb for each Gli2 locus from the bread wheat cultivar Chinese Spring. They highlighted 12 intact α-gliadin genes, among which 9 were expressed. Wang et al. [19] and Cho et al. [20] found higher numbers of active genes: they respectively showed that the Xiaoyan 81 bread wheat cultivar displayed 25 intact expressed α-gliadin sequences through a third-generation RNA sequencing technique and that the Keumkang bread wheat cultivar flour contained 27 different α-gliadins through a proteomic approach. The α-gliadin proteins display four main T-cell stimulatory epitopes involved in CD: the major DQ2.5-glia-α1 and DQ2.5-glia-α2 (P{F/Y}PQPQLPY and PQPQLPYPQ, respectively) and the minor DQ2.5-glia-α3 and DQ8-glia-α1 epitopes (FRPQQPYPQ and QGSFQPSQQ, respectively). In the α-gliadins from the D genome, the DQ2.5-glia-α2 epitope can be displayed in one, two or three copies and leads, when three copies are present, to the most immunogenic fragment of α-gliadin sequences, known as the 33-mer fragment [21, 22].

Each of the four main α-gliadin T-cell stimulatory epitopes involved in CD can be displayed in its canonical form recognized by the immune system of CD patients. However, allelic variants of these epitopes, resulting from amino acids substitution or deletion, also exists among the α-gliadin genes. Interestingly, these allelic variants have a reduced or suppressed immunogenicity [23]. Several techniques have been developed to measure the toxic potential held in the cereal grains. The first one is the enzyme-linked immunosorbent assay (ELISA), which mainly exists in the form of test kits to quantitate the amount of gluten in food samples and detect gluten contamination. These kits are based on different antibodies which have been raised against gluten proteins [24]. However, the restricted specificity of the antibodies regarding canonical epitopes limits the accuracy of ELISA tests to study the immunogenic potential of varieties. Amaya-Gonzalez et al. [25] developed another technique based on aptamers to detect the 33-mer fragment. This tool allows quantifying the gluten content through an electrochemical competitive enzyme-linked assay on magnetic particles with a six-fold improved sensitivity compared to the ELISA reference test. Nevertheless, these aptamers do not focus on individual epitopes and the absence of cross-reactivity with allelic variants of the 33-mer has not been demonstrated. Two other strategies have recently been developed and make possible discriminating canonical epitopes from their allelic variants: liquid chromatography combined to mass spectrometry (LC-MS) on the one hand [26–28], and TaqMan probes on the other [29]. The LC-MS technique is designed to study the epitope composition at the protein level, whereas TaqMan probes focus on α-gliadin transcripts. A relationship was observed between the results of Van den Broeck et al. [28], who developed a LC-MS method, and those of Salentijn et al. [30] who carried out a high throughput RNA sequencing of α-gliadin sequences coming from the same accession.

Several possibilities are currently being investigated to address the CD problem, like gluten detoxification, modulation of mucosal permeability, antigen presentation blockade, raising monoclonal antibodies against inflammatory cytokines, inhibition of T-cell recruitment or oral tolerance induction [31]. Among them, the development of new cereal varieties that lack immunogenic peptide, but still display good baking characteristics, is a promising approach [11, 31]. Indeed, bread wheat and related taxa display a high genetic variability which can be exploited. Among them, spelt is also a member of the *Triticum aestivum* species. It displays interesting features such as its adaptability to poor soils, harsh climatic conditions and low-input tolerance. Moreover, spelt germplasm collections are characterized by a high genetic diversity [32–35]. Illustrations of this variability were observed for features such as immunogenic potential, bread-making qualities and content in proteins, lipids, micronutrients and fibers [32, 35–38]. Furthermore, tools used to study the CD-related immunogenic potential of bread wheat can also be used for spelt since it displays the same epitope allelic variants [35].

Whereas bread wheat has already been broadly studied for its CD-related content, only a few publications has approached it for spelt, and never with a plant collection representative of its large diversity. Ribeiro et al. [39] and Escarnot et al. [40] highlighted higher reactivities for spelt accessions than for bread wheat ones while Gélinas and McKinnon [41] did not observe any significant difference. However, these three works did not study the immunogenic content strictly speaking given that they used ELISA kits which detect both canonical epitopes and allelic variants. Ozuna et al. [17] performed a high throughput sequencing of the 5′ end of α-gliadin sequences from 16 bread wheat and 11 spelt accessions and they showed that the expression profile of the 33-mer peptide was similar in both sub-species. Ozuna and her colleagues [42] also analyzed the occurrence of the four major T-cell stimulatory epitopes among the α-gliadin sequences reported on the NCBI database and they found comparable frequencies in spelt and bread wheat sequences. Schalk et al. [26] and Prandi et al. [43] used a LC-MS strategy to specifically quantify the immunogenic content of spelt and bread wheat accessions. Among the four major α-gliadin CD-related epitopes studied in this work, Prandi et al. [43] quantified the expression of the DQ2.5-glia-α3 epitope whereas Schalk et al. [26] focused on the 33-mer peptide. In both studies, spelt and bread wheat accessions did not set apart from each other. Thus, the few available studies enabling to compare the immunogenic content of spelt to the one of bread wheat does not seem to indicate major differences between these two subspecies. However, spelt germplasm collections display a high level of genetic diversity, which has never been studied in the framework of celiac disease.

Beyond the search for non- or low-immunogenic accessions, attention should be also paid to the putative influence of cultivation techniques on the amount of immunogenic sequences expressed. Among the factors influencing the expression of gluten proteins, the nitrogen (N) fertilization strategy has been identified as an important component [44–46]. The α-gliadins being a sulfur (S)-rich prolamin protein class, their expression has also been shown to be affected by the S nutrition in einkorn grains [47]. Several studies have shown that the N rates applied influenced the expression of α-gliadins [48–50]. The expression of the four main α-gliadin epitopes involved in CD could thus be affected by the fertilization strategy since similar trends between the global expression of α-gliadins and the amount of epitope transcripts have been previously observed [29]. Consequently, it would be relevant to investigate this hypothesis and analyze whether an appropriate fertilization strategy could have a beneficial impact in reducing the amount of expressed epitopes.

The objective of this work was to study the potential of spelt regarding the immunogenicity related to CD as a function of both genetic and environmental factors. This was carried out by (i) analyzing the expression profile of the four main α-gliadin T-cell stimulatory epitopes in a wide collection of spelt accessions using epitope-specific TaqMan probes, (ii) analyzing the correlation between this expression and qPCR results from gDNA samples, and (iii) investigating whether environment and crop practices could have an impact on this expression by testing its stability over consecutive years and comparing different N fertilization treatments.

## Results

### Epitope expression profiling in a wide set of spelt accessions

**Fig. 1:**
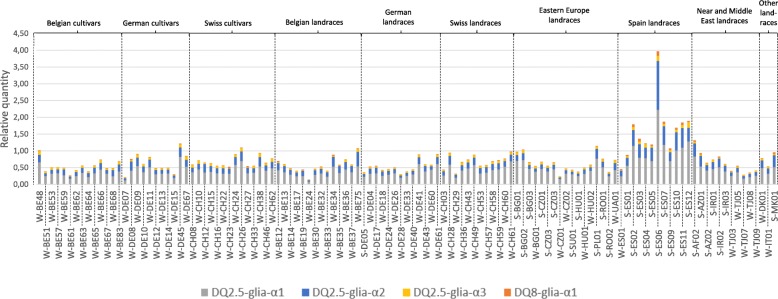
Epitope expression profile of 121 spelt cultivars and landraces from different geographical provenances. Epitope expression levels were measured through the use of specific TaqMan probes targeting the canonical form of the four main α-gliadin epitopes involved in CD. The relative quantities were calculated by dividing 2^ΔCt^ values by a normalization factor obtained through the expression analysis of stable reference genes

**Fig. 2:**
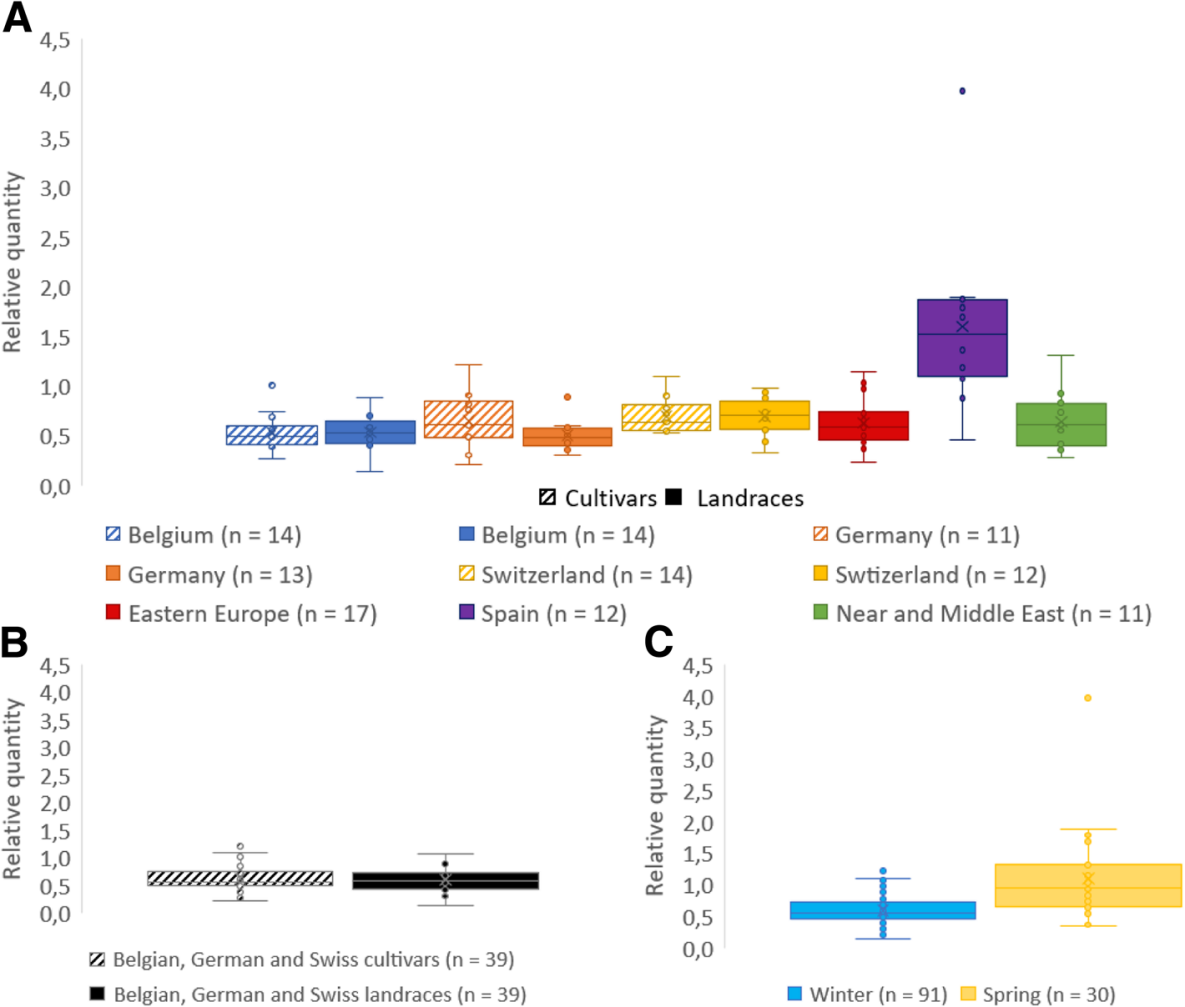
Relative quantities of epitope transcripts in different subsets of spelt cultivars and landraces. These subsets are divided according to (**a**) their geographical provenance, (**b**) their breeding status, and (**c**) their habit

The expression levels of the four main canonical epitopes involved in CD were determined on 121 spelt accessions, including cultivars and landraces from Northwestern Europe (Belgium, Germany, Switzerland) as well as landraces from Eastern Europe, Spain and Near and Middle East (Fig. 1). A very high variability in the epitope expression levels was revealed with cumulated relative quantities ranging from 0.14 for the Belgian accession W-BE24 to 3.97 for the Spanish accession S-ES06. Spanish accessions globally displayed the highest epitope expression levels (Fig. 2a) and the analysis of variance revealed very highly significant differences between accession groups (*P* = 2.666e-11).

The putative effect of breeding strategies on the amount of CD-related epitope transcripts was investigated by comparing epitope expression levels measured on cultivars and landraces from a same geographical provenance, i.e. Belgium, Germany and Switzerland (Fig. 2a and b). No statistical difference was found in expression level either between cultivars and landraces taking all three countries together (*P* = 0.576, Fig. 2b), or between cultivars and landraces taking each country separately (Belgium: *P* = 0.63, Germany: *P* = 0.248, Switzerland: *P* = 0.993) (Fig. 2a).

The influence of the habit, i.e. winter-sown or spring-sown accessions, was studied by comparing epitope expression levels from all winter spelt accessions against those of all spring accessions (Fig. 2c). This resulted in a very highly significant difference (*P* = 2.634e-8) between these two groups, the spring accessions displaying higher epitope expression levels than the winter ones. In addition, this trend was also observed within sub-groups representing geographical provenances, i.e. Eastern Europe, Spain and Near and Middle East.

### Quantitative PCR on gDNA samples and correlation with epitope expression

Measures of the gDNA epitope content were carried out on a sub-group of accessions representative of geographical provenance, breeding status and epitope expression levels. Epitope relative quantities measured on gDNA samples ranged from 0.11 for the Belgian cultivar W-BE48 to 0.63 for the German landrace W-DE28 (see Additional file 1). The comparison of the accession habit showed that winter accessions displayed a significantly higher genomic epitope content (0.37 ± 0.12) than the spring ones (0.21 ± 0.04) (*P* = 5.286e-4). However, no significant correlation was found between cDNA and gDNA results (*r* = − 0.222; *P* = 0.14). Moreover, spring accessions displayed higher epitope expression levels but lower genomic epitope occurrence than winter ones.

### Interannual epitope expression analysis

**Fig. 3:**
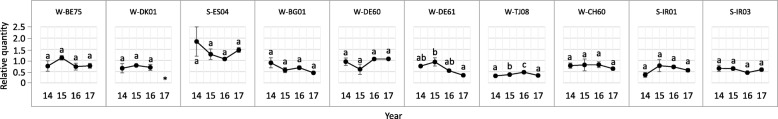
Epitope expression profile of 10 contrasted spelt accessions harvested in 2014, 2015, 2016 and 2017. Inter-annual significant differences for each accession are denoted by different letters. Data are presented as mean ± standard deviation. *: Missing data

The interannual variations of the epitope expression levels were investigated with samples harvested in 2014, 2015, 2016 and 2017 for 10 contrasted spelt accessions previously studied [29, 35] (Fig. 3). A two-way ANOVA revealed a significant effect for the accessions, but none for the harvest years. However, even if the accession effect seems to have a strong influence on the epitope expression (*P* = 8.844e-13), a significant interaction between the accession and harvest year factors was also pointed out (*P* = 2.322e-3, see additional file 2). The significance of these two factors cannot thus be undoubtedly assessed.

No significant interannual variation was found for eight accessions while slight variations were observed for the W-DE61 and W-TJ08 accessions (*P* = 1.445e-2 and 8.341e-5, respectively). As shown in Fig. 3, the general ranking of the accessions was conserved from one harvest year to another, with the Spanish S-ES04 and the Tajik W-TJ08 accessions showing the highest and the lowest epitope expression levels, respectively. A deeper analysis revealed significant differences in the epitope expression, whatever the harvest year.

### Epitope expression according to harvest date and N fertilization

**Fig. 4:**
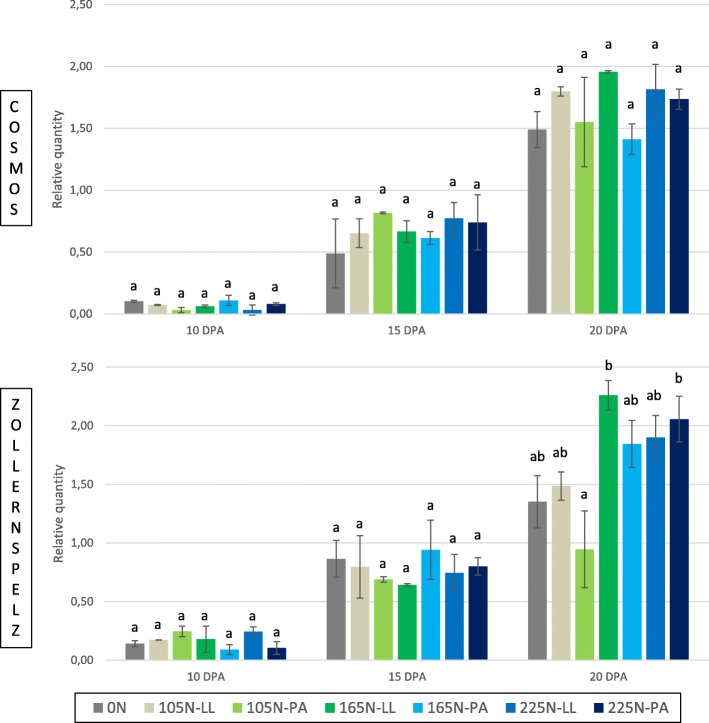
Epitope expression levels measured after different N fertilization strategies for Cosmos and Zollernspelz spelt cultivars. Seven N modalities were tested and included one control without N application (0 N) and three increasing N amounts (105, 165 and 225 kg N/ha), fractionated into three application periods. The last application was carried out either at the last leaf stage (LL) or the post anthesis stage (PA). The expression levels were studied on seed samples harvested at 10, 15 and 20 DPA. Within each of these harvest modalities, significant differences are denoted by different letters. Data are presented as mean ± standard deviation

The epitope expression analysis showed very highly significant differences between samples harvested 10, 15 and 20 days post-anthesis (DPA) for both studied spelt cultivars Cosmos (W-BE56) and Zollernspelz (W-DE11) (*P* = 2.2e-16 and *P* = 3.437e-16, respectively), with epitope expression levels increasing from early to later harvest dates (Fig. 4).

For Cosmos, no statistical difference appeared between modalities of N fertilization, neither for samples harvested 10 DPA (*P* = 0.073), 15 DPA (*P* = 0.485) or 20 DPA (*P* = 0.124).

For Zollernspelz at 20 DPA, the treatment modalities 165 N-LL and 225 N-PA (i.e. total amount of N applied of 165 and 225 kg/ha with the last of the three applications carried out at the last leaf and the post anthesis stage, respectively) gave rise to a significantly higher epitope expression compared to the one of the modality 105 N-PA, which yielded the lowest value (*P* = 4.31e-3). Conversely, no difference between modalities was detected for samples collected at 10 DPA (*P* = 0.13) and 15 DPA (*P* = 0.619).

## Discussion

### CD-related epitope expression in a wide spelt collection

The main objective of this work was to gain insight into the potential of spelt regarding its immunogenicity for CD patients. To reach this objective, a collection of 121 spelt accessions, representative of different geographical provenances, status (cultivar and landrace) and habits (winter and spring) was assembled and their expression profile of CD-related epitopes was evaluated.

The profiling of the epitope expression among the 121 spelt accessions revealed very high variations, with a factor of 28.4 between the lowest and the highest epitope expression level measured, which is in line with the high level of spelt genetic diversity previously reported according to microsatellite markers [33], glutenin subunit variation [32, 34] and variance in agronomic traits [51]. Highly significant differences between accession provenances were highlighted. The Spanish and Tajik accessions stood out from the others by, respectively, high and low amounts of epitope transcripts measured for each accession of these provenances. This feature can be linked to the work of Bertin et al. [33]. Studying the genetic diversity and structure of the spelt gene pool by using microsatellite markers, they showed that almost all Spanish and Tajik accessions clustered together in two distinct groups which clearly set them apart from spelts of other geographical provenances. In addition, these results are also in line with previous studies which focused on only one accession of each geographical provenance [29, 35].

The main spelt breeding programs in the world have been led in Belgium, Germany and Switzerland, with official research programs starting around 1950. The epitope expression levels of cultivars and landraces from these countries were compared to each other to highlight a putative effect of spelt breeding on CD immunogenicity. The statistical analysis did not show any difference between these two groups. Thus, the selection carried out in spelt breeding programs do not seem to influence the epitope expression. This could agree with the results obtained on wheat by Kasarda et al. [52], who showed that breeding during the twentieth century did not increase the gluten content in the United States. However, the results obtained by Kasarda et al. [52] and in this study must be compared with caution since the gluten content does not provide direct information about the α-gliadin immunogenic content. More recently, Schalk et al. [26], using a liquid chromatography tandem mass spectrometry assay, did not show any higher amount of 33-mer peptide in modern versus old wheat cultivars. In contrast, Ribeiro et al. [39] showed in ELISA assays that landraces presented a higher reactivity than varieties in bread wheat. However, they used the R5 monoclonal antibody, which is normally used to quantitate the amount of gluten in food samples and is not specific to CD-related epitopes, thus revealing both immunogenic and non-immunogenic epitopes altogether.

The analysis of expression data according to habit, i.e. winter or spring spelt, showed that spring accessions displayed on average a higher amount of epitope transcripts than the winter ones, whether provenances were analyzed together or separately. These differences in the epitope expression can have two explanations. Firstly, they could be linked to the differences in the gene pools used in breeding programs for spring versus winter accessions. Secondly, they could also be attributed to the differential environmental effect due to the sowing date (October versus March, respectively). The epitope expression levels may indirectly give an idea of the α-gliadin expression, as shown in Dubois et al. [29], and could thus be linked to the bread-making properties. Interestingly, Maghirang et al. [53], comparing spring and winter bread wheat accessions, showed that the grains and flour of spring accessions displayed better bread-making qualities than winter ones. In the context of CD, if these higher quality properties go hand in hand with higher immunogenicity, it thus means that it would be more interesting to work with winter spelt varieties rather than spring ones – which is already the case in spelt breeding countries like Belgium, Germany and Switzerland – to develop low celiac potential accessions.

### Evaluation of the putative correlation between expressed and genomic epitope sequences

The aim was to investigate the relation between the genomic occurrence of sequences encoding for immunogenic epitopes and their expression. In addition, this comparison also allowed analyzing whether the epitope-specific TaqMan probes could be used on gDNA, which would be simpler and cheaper than on cDNA, to have an accurate idea of accession immunogenicity. The qPCR results obtained from gDNA samples of 45 accessions were compared to those derived from cDNA samples. Relative quantities measured showed a factor of 28.4 (from 0.14 to 3.97) and 5.7 (from 0.11 to 0.63) between the lowest and the highest values for cDNA and gDNA samples respectively. This indicates that the use of epitope-specific TaqMan probes on gDNA leads to an underestimation of the variability in epitope content among the accessions. Moreover, the analysis of variance revealed an absence of correlation between these two sets of data. Thus, when aiming at evaluating the immunogenicity of an accession, the use of epitope-specific TaqMan probes on gDNA does not constitute a valid alternative to the epitope expression study.

The absence of correlation between the two sets of data (cDNA versus gDNA) is striking but could be explained by the multigenic character of the α-gliadin family and the abundance of pseudogenes. Previous studies pointed out the high and variable number of α-gliadin coding sequences held in the haploid genome of bread wheat, ranging from 25 [54] to 150 [55], depending on the variety. Among these coding sequences, it was demonstrated by Anderson and Greene [16] that about half of them were pseudogenes, due to the presence of at least one premature stop codon (PSC). More recently, Ozuna et al. [17] found pseudogenes proportions of 39, 76 and 63% in the genomes of diploid, tetraploid and hexaploidy wheat species, respectively. When measuring the genomic epitope content with the TaqMan probe/primer systems used in this work, pseudogenes are also detected, unless the PSC is located in the epitope sequence targeted by the TaqMan probe or the primer hybridization site. On the contrary, pseudogenes are practically absent in cDNA samples [35] and are thus not detected when studying the epitope expression profile on the cDNA. This may explain the absence of correlation highlighted in this study between the occurrence of α-gliadin epitope sequences in the spelt genome and their expression levels.

Despite the absence of correlation between qPCR values from gDNA and cDNA samples, spring spelt accessions displayed a significantly lower occurrence of epitope sequences in their genome, while their expression of epitope transcripts was significantly higher than in winter spelts. This could be explained by two hypotheses: either the putative higher amount of pseudogenes in winter spelt accessions and/or the spring environmental conditions that would favor the epitope expression. These observations underline the complexity to lead breeding strategies when dealing with the CD problem and demonstrate the need to work with molecular markers focusing on epitope transcripts rather than on genomic sequences.

### Influence of harvest year and impact of N fertilization

It has already been shown that environmental conditions, and thus the harvest year, can influence gluten composition in bread wheat [26, 56] and that the N fertilization strategy (amount and splitting) can impact the grain protein content and the relative abundances of the different seed storage proteins [45, 46, 48].

To investigate whether the harvest year could have an impact on the immunogenicity level, the expression of the four major α-gliadin canonical epitopes was evaluated in 10 contrasted accessions harvested during four consecutive years. This revealed a significant effect of the accessions on the epitope expression levels, whereas no impact of the harvest year was observed. This contrasts with the results of Schalk et al. [26] who showed that the harvest year had a significant influence on the 33-mer content in four bread wheat cultivars grown at the same location in Hungary and harvested in 2011, 2012 and 2014. The authors even suggested that the environmental factor had a greater influence on the 33-mer content than the genetic background of the four wheat cultivars. However, these results are not really comparable since the years and locations differ; these discrepancies could be partly attributed to different growing conditions as Hungary’s continental climate may lead to periods of drought more frequently. The genotype x environment interaction highlighted in this work could explain these contradictory results as well. In addition, these discrepancies could also find an explanation in the fact that the traits quantified in the present study and in the one of Schalk et al. [26] are not the same (expression of the four main α-gliadin epitopes vs 33-mer, respectively).

Interestingly, the analysis of climatic conditions for each year in Gembloux during the grain filling period revealed some differences (see additional file 3): whereas the climatic conditions have been by far the wettest during the 2016 grain filling period, climatic data for 2017 display the opposite trend with a higher mean temperature and a low level of rainfalls. Despite these fluctuating meteorological conditions, no significant differences in the epitope expression levels have been pointed out according to the harvest year.

However, the statistical analysis still highlighted an interaction between the accession effect and the harvest year. This means that, even if the harvest year parameter taken alone does not have a significant influence, the epitope expression evolution from 1 year to another is not the same for each accession, some accession/harvest year combinations leading to significantly different epitope expressions levels than others.

The influence of N fertilization on the amount of epitope transcripts was evaluated on a Belgian and a German spelt cultivar, i.e. Cosmos and Zollernspelz, respectively. Seven N modalities, differing by the N amount and splitting, were applied. This split application of N fertilizer has been pointed out as an effective way of improving wheat protein content [44]. Xue et al. [46] showed that N splitting changes the grain protein composition by increasing the proportions of gliadins and glutenins, leading to an improved baking quality of wheat flour. They even postulated that N splitting is a more effective way to improve wheat quality than the increase in the N applied, offering the potential to reduce the amount of N fertilizer.

The highest epitope expression levels were reached at 20 DPA, whatever the accession or the N treatment modality. This is in accordance with the α-gliadin expression peak previously observed around 20 DPA in bread wheat [57]. The analysis of variance revealed a slight effect of the fertilization strategy on the amount of epitope transcripts measured at 20 DPA for Zollernspelz, but not for Cosmos. Wieser and Seilmeier [45] also noticed that the degree of N fertilization effects on the quantities and proportions of flour protein groups were strongly dependent on the variety. The absence of difference in epitope expression for Cosmos cultivar can be linked to the work of Garcia-Molina and Barro [50] who worked with the bread wheat cultivar Bobwhite and showed that the application of increasing amounts of N fertilizer did not modify the α-gliadin content for some fertilizing strategies. Bonnot et al. [47] also highlighted that the quantity of α-gliadins in einkorn grains was not significantly modified by the N nutrition; however, their synthesis was affected by the S nutrition. For Zollernspelz, the global trend – i.e. increasing epitope expression when raising the N fertilization – is in line with the accumulation of gliadins and total prolamin content when increasing N fertilization, as reported in several studies [46, 58, 59].

## Conclusions

The development of new varieties lacking immunogenic peptides is one of the strategies currently investigated to face the CD issue. The knowledge of available diversity is a crucial information for breeding purposes. This work provides useful information about the diversity held in a wide collection of spelt cultivars and landraces from different geographical provenances, in terms of CD-related epitope expression levels. Some accessions were shown to display a low CD-related content, but they cannot be safely consumed by celiac patients since even low amounts of immunogenic epitopes still stimulate the immune system. This work, however, provides important knowledge about the available plant material which could be mobilized in breeding programs combining traditional and new promising molecular approaches. A correlation analysis revealed no correlation between the epitope contents measured on cDNA and gDNA samples of identical accessions. This could be linked to the multigenic character of the α-gliadin family and the high number of pseudogenes, making conventional breeding alone probably not efficient enough to develop celiac-safe varieties. However, several promising molecular approaches are currently being investigated in bread wheat or barley, like RNA interference strategies with hairpin RNA constructs [60, 61] or antisense constructs [62], and the generation of deletion lines lacking loci encoding for α-gliadins [63]. In addition, new genome editing technologies have been shown to be efficient to significantly reduce the α-gliadin immunogenicity [64, 65]. The epitope expression stability was evaluated by harvesting seeds during four consecutive years and by studying the influence of different N fertilization strategies. Even if an interaction between the accession and harvest year effects was pointed out, the environmental factors investigated did not seem to have a major impact on the epitope expression. Consequently, lowering the CD-related epitope content by modifying environmental conditions and/or applying a specific method of N fertilization could be inefficient. However, the genotype x environment interaction does not enable to draw definitive conclusions and it would thus be interesting to study further the environmental influence on the expression of CD-related epitopes.

## Methods

### Plant materials and field trials

A collection of 121 spelt accessions was constituted with grains obtained from various sources, namely: the Agroscope (Nyon, Switzerland), the Center for Genetic Resources (Wageningen, The Netherlands), the Walloon Agricultural Research Center (Gembloux, Belgium), the Crop Research Institute (Prague, Czech Republic), the Institute of Plant Genetics and Crop Plant Research (Gatersleben, Germany), the United States Department of Agriculture (Washington, USA) and the Vavilov Institute of Plant Genetic Resources (Saint Petersburg, Russia) (Additional file 4). This collection included cultivars and landraces from the three main spelt breeding countries (Belgium, Germany and Switzerland) and landraces from different provenances in Europe and Asia, which were studied for their CD-related content. This panel included ten contrasted spelt accessions previously selected as representative of spelt diversity [35] and used to validate the development of epitope-specific TaqMan probes [29]. All these accessions were grown under field conditions in Gembloux (Belgium) in 2015. (Additional file 3). The growing conditions did not include any pest control and each plot was 1.3 m in length, 0.4 m in width and consisted in two ear-rows. On the basis of N residuals measured in the soil, the amount of N fertilizer to apply each year to the crop was calculated with the Azobil software (INRA, Laon, France), with the aim of reaching 150 kg/ha of available N. In order to investigate the interannual variation of the epitope expression, the 10 contrasted spelt accessions mentioned above were grown in the field in Gembloux during 4 years from 2014 to 2017 (Additional file 3). For epitope expression analyses, the ears were enclosed with cellophane bags to ensure self-pollination and immature grains were harvested 20 days post-anthesis (DPA), immediately frozen in liquid nitrogen and stored at − 80 °C. For studies focusing on genomic DNA (gDNA), shoots were collected after the immature grain harvest, frozen in liquid nitrogen and also stored at − 80 °C.

The trial focusing on the putative influence of the nitrogen (N) fertilizer amount on the epitope expression was also carried out in Gembloux (Belgium) in 2017 and included two spelt cultivars widely cultivated in Belgium and Germany, i.e. Cosmos (W-BE56) and Zollernspelz (W-DE11) respectively. The growing conditions included a fungicide and a growth regulator treatment. The experiment was designed as a randomized complete block consisting of two blocks and seven applied N modalities. These modalities included one control without N application and three increasing N amounts, aiming at reaching respectively 105, 165 and 225 kg/ha of available N, on the basis of N residuals measured in the soil. The applied N was fractionated into three application periods. The first and second applications were at tillering and first node stages, respectively, and the last application was carried out either at the last leaf stage (LL) or the post anthesis stage (PA). Immature grains were harvested for each modality at 10, 15 and 20 DPA.

### RNA extraction, cDNA synthesis and gDNA extraction

Total RNA was extracted from 100 mg of ground seeds using the NucleoSpin® RNA Plant kit (Macherey-Nagel, Germany) and quantified by spectrometry. First-strand complementary DNA (cDNA) was synthetized from 200 ng RNA using the RevertAid H Minus First Strand cDNA Synthesis Kit (Thermo Scientific) with oligo(dT)_18_ primer in a volume of 20 μl. The cDNA was then quantified by spectrometry. The gDNA was extracted from 100 mg of ground spelt shoot following a modified version of the Doyle protocol [66]: the 2/3 volumes of cold isopropanol were replaced by the addition of NaCl 2 M and 2 volumes of ethanol. The wash buffer was replaced by ethanol 70% and the protocol was terminated by directly storing the samples at − 20 °C after RNase incubation during 30 min at 37 °C. The absence of RNA contamination was checked by running gDNA samples on a 1% agarose gel. The extracted DNA was further quantified by spectrometry.

### Expression of the four α-gliadin CD-related canonical epitopes

Primers and TaqMan probes previously developed to study the expression of the four α-gliadin canonical epitopes involved in CD (DQ2.5-glia-α1, −α2, −α3 and DQ8-glia-α1) and to normalize their expression to the one of stable reference genes [29] were used (Additional file 5). The three most stable reference genes, i.e. ADP-ribosylation factor (ARF), similar to RNase L inhibitor-like protein (RLI) and a protein of unknown function [DUF52 family] (DUF), were selected as the most appropriate number of genes to normalize epitope expression values, taking into account calculation accuracy and technical considerations. The primers targeting reference genes were adapted from those designed by Paolacci et al. [67].

Quantifications were carried out using 10 μl of Takyon™ No Rox Probe 2× Mastermix dTTP Blue (Eurogentec, Belgium), 300 nM of each primer, 100 nM of TaqMan probe labeled with the FAM fluorophore and TAMRA quencher (Eurofins Genomics, Germany), 2 μg of cDNA and nuclease-free water (Thermo Scientific) for a total volume of 20 μl. Two biological replicates of each sample, i.e. coming from 2 plants grown in the same plot, were loaded in triplicate in a Hard-Shell® 96-Well PCR skirted white plate and sealed with a Microseal® ‘B’ PCR Plate Sealing Film (Bio-Rad). PCR amplifications were performed by the C1000 Touch™ Thermal Cycler coupled to the CFX96™ Real-Time detection system (Bio-Rad). The following thermal cycling protocol was used: initial denaturation at 95 °C for 3 min followed by 40 cycles at 95 °C for 10 s and 69 °C for 1 min. The epitope expression levels were calculated following the 2^ΔCt^ method detailed in Dubois et al. [29]. All qPCR datasets generated in this study are reported in additional file 6.

### Quantitative PCR on gDNA samples

With the aim of investigating whether epitope-specific TaqMan probes could directly be used on gDNA, the correlation between the epitope genomic occurrence (gDNA) and their expression (cDNA) was studied. A sampling among the accessions was thus carried out to study their gDNA epitope content. Based on epitope expression levels measured on the 121 spelt accessions harvested in 2015, five accessions were selected for each of the nine sub-groups (i.e. Belgian cultivars, German cultivars, Swiss cultivars, Belgian landraces, German landraces, Swiss landraces and landraces from Eastern Europe, Spain and Near and Middle East). Within each sub-group, the five selected accessions consisted in those displaying the highest (2), the lowest (2) and a mean (1) epitope expression level. Four hundred ng gDNA were used to quantify the gDNA sequences coding for the four α-gliadin canonical epitopes involved in CD (DQ2.5-glia-α1, −α2, −α3 and DQ8-glia-α1), using the same primers, TaqMan probes and conditions as above.

Results could not be normalized thanks to reference genes since these genes were selected as stable reference genes based on their expression rather than on the genomic occurrence. Consequently, strict spectroscopy measurements were made to ensure that the gDNA used as template in qPCR reactions was present in equal amounts in all the samples. These measures were carried out with a NanoDrop One^c^ spectrophotometer (Thermo Scientific), which provides, in addition to optical densities and DNA concentrations, full-spectral data with information about the sample quality, and putative contaminants. For each sample, the gDNA concentration was measured in triplicate.

### Data analysis

The deviations between expression values for biological replicates were reported as standard deviations. The significance of the observed differences (*P* < 0.05) was assessed through ANOVA tests using the *lm* function (normal linear models) and time was considered a fixed factor. Multiple comparison tests (Tukey Contrasts) were performed thanks to the *glht* function. The resulting *p*-values were adjusted for multiple comparisons by a Bonferroni correction. The putative correlation between results obtained from cDNA and gDNA samples was evaluated using the non-parametric Spearman’s rank correlation test and the *cor.test* function. All statistical analyses were performed in R (R Development Core Team). All assumptions were checked graphically.

## Additional files

Additional file 1: Relative quantities of the four cumulated α-gliadin epitopes involved in CD measured on gDNA samples with TaqMan probes targeting only the canonical form of these epitopes. The file presents the genomic occurrence of the four main α-gliadin T-cell stimulatory epitopes involved in CD in a set of 45 spelt accessions. (PDF 258 kb)

Additional file 2: Two-way analysis of variance for the epitope expression analysis of 10 contrasted spelt accessions during 4 consecutive years. The file presents the two-way ANOVA carried out on the results of the analysis investigating the influence of the harvest year on the expression of the four α-gliadin CD-related epitopes. (DOCX 12 kb)

Additional file 3: Cultivation conditions in which the spelt accessions studied in this work have been grown. This file provides information about cultivation conditions, i.e. geographic coordinates, altitude, soil type, previous crop, climate type, mean temperature and rainfalls, in which spelt accessions have been grown. (DOCX 13 kb)

Additional file 4: Spelt accessions used to study the α-gliadin canonical epitope expression with TaqMan probes. The file details the breeding status, the geographical provenance, the code, the year of release, the number and the name of the 121 spelt accessions studied in this work. (XLSX 18 kb)

Additional file 5: List of the primers and probes used to measure the expression levels of the four α-gliadin major T-cell stimulatory epitopes in their canonical form and the expression levels of reference genes. The file presents the primers and probes used in this study for qPCR experiments. (XLSX 11 kb)

Additional file 6: Averaged Ct values measured for each biological replicate of the samples studied in this work. The file displays all the Ct values measured during qPCR experiments. (XLSX 43 kb)

## Abbreviations

ANOVA: Analysis of Variance
ARF: ADP-Ribosylation Factor
BAC: Bacterial Artificial Chromosome
CD: Celiac Disease
cDNA: Complementary Deoxyribonucleic Acid
DPA: Day Post-Anthesis
DUF: protein of unknown function [DUF52 family]
ELISA: Enzyme-Linked Immunosorbent Assay
FAM: Fluorescein Amidite
gDNA: Genomic Deoxyribonucleic Acid
HMW-GS: High Molecular Weight Glutenin Subunit
LC-MS: Liquid Chromatography-Mass Spectrometry
LL: Last Leaf
LMW-GS: Low Molecular Weight Glutenin Subunit
N: Nitrogen
PA: Post-Anthesis
PSC: Premature Stop Codon
qPCR: Quantitative Polymerase Chain Reaction
RLI: similar to RNase L Inhibitor-like protein
RNA: Ribonucleic Acid
S: Sulfur
TAMRA: Carboxytetramethylrhodamine
