# Supplementary material for: Genetic and environmental factors affecting the expression of α-gliadin canonical epitopes involved in celiac disease in a wide collection of spelt (Triticum aestivum ssp. spelta) cultivars and landraces
Source: BMC Plant Biol. 2018 Nov 1;18:262. doi: 10.1186/s12870-018-1487-y (PMC6211434; doi:10.1186/s12870-018-1487-y)
Supplement: Supplementary file 1 — Relative quantities of the four cumulated α-gliadin epitopes involved in CD measured on gDNA samples with TaqMan probes targeting only the canonical form of these epitopes. The file presents the genomic occurrence of the four main α-gliadin T-cell stimulatory epitopes involved in CD in a set of 45 spelt accessions. (PDF 258 kb) [file 12870_2018_1487_MOESM1_ESM.pdf]

Additional file 1. Relative quantities of the four cumulated α-gliadin epitopes involved in CD measured on gDNA samples with TaqMan probes targeting only the canonical form of these epitopes.

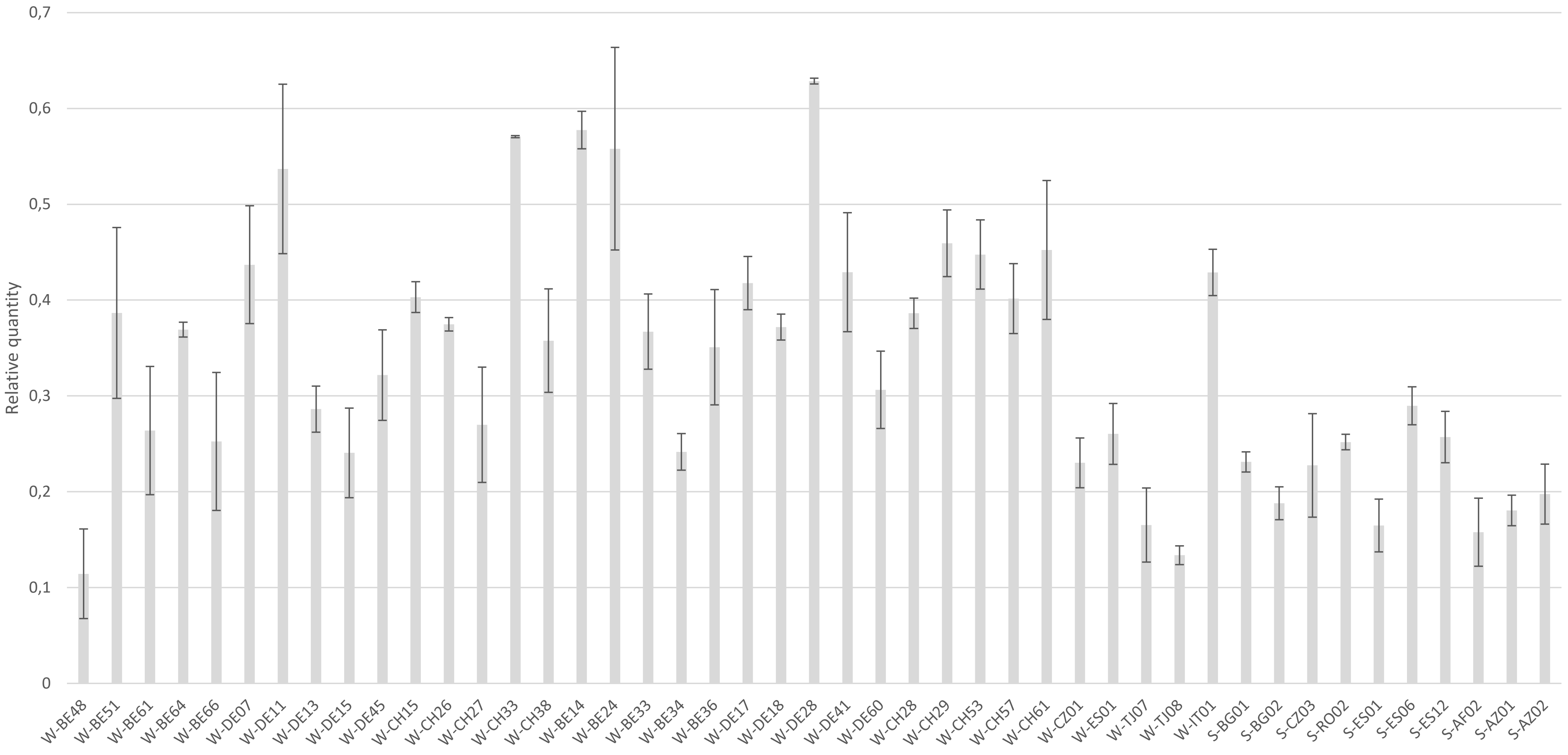

On the basis of the epitope expression levels measured for 120 spelt accessions (Figure 1), five accessions were selected for each of the nine sub-groups (i.e. Belgian cultivars, German cultivars, Swiss cultivars, Belgian landraces, German landraces, Swiss landraces and landraces from Eastern Europe, Spain and Near and Middle East) to study their gDNA epitope content. These results were then compared to those obtained on cDNA samples to investigate whether a correlation could be assessed between these two sets of data. Data are presented as mean ± standard deviation.
