# Supplementary material for: Genetic and environmental factors affecting the expression of α-gliadin canonical epitopes involved in celiac disease in a wide collection of spelt (Triticum aestivum ssp. spelta) cultivars and landraces
Source: BMC Plant Biol. 2018 Nov 1;18:262. doi: 10.1186/s12870-018-1487-y (PMC6211434; doi:10.1186/s12870-018-1487-y)
Supplement: Supplementary file 2 — Two-way analysis of variance for the epitope expression analysis of 10 contrasted spelt accessions during 4 consecutive years. The file presents the two-way ANOVA carried out on the results of the analysis investigating the influence of the harvest year on the expression of the four α-gliadin CD-related epitopes. (DOCX 12 kb) [file 12870_2018_1487_MOESM2_ESM.docx]

**Additional file 2. Two-way analysis of variance for the epitope expression analysis of 10 contrasted spelt accessions during 4 consecutive years.**

| Source of variation | MS | *d.f.* | *F* | *P* |
| --- | --- | --- | --- | --- |
| Spelt accessions | 0.618 | 9 | 22.471 | 8.844e-13 |
| Year | 0.051 | 3 | 1.859 | 0.153 |
| Spelt accessions* Year | 0.075 | 26 | 2.718 | 2.322e-3 |
