# Supplementary material for: Genetic and environmental factors affecting the expression of α-gliadin canonical epitopes involved in celiac disease in a wide collection of spelt (Triticum aestivum ssp. spelta) cultivars and landraces
Source: BMC Plant Biol. 2018 Nov 1;18:262. doi: 10.1186/s12870-018-1487-y (PMC6211434; doi:10.1186/s12870-018-1487-y)
Supplement: Supplementary file 3 — Cultivation conditions in which the spelt accessions studied in this work have been grown. This file provides information about cultivation conditions, i.e. geographic coordinates, altitude, soil type, previous crop, climate type, mean temperature and rainfalls, in which spelt accessions have been grown. (DOCX 13 kb) [file 12870_2018_1487_MOESM3_ESM.docx]

**Additional file 3: Cultivation conditions in which the spelt accessions studied in this work have been grown.**

| **Year** | **Plot** | | | | **Climate** | | |
| --- | --- | --- | --- | --- | --- | --- | --- |
|  | **Geographic coordinates** | **Altitude (m)** | **Soil type** | **Previous crop** | **Climate type** | **Mean temperature (°C)²** | **Rainfalls (mm)²** |
| 2014 | 50.558596N, 4.716483E | 166 | Silty | Sugar beet | Temperate oceanic | 15.4 | 65.9 |
| 2015 | 50.568452N, 4.741172E | 161 | Silty | Pea | Temperate oceanic | 15.3 | 47.2 |
| 2016 | 50.560465N, 4.711088E | 167 | Silty | Rapeseed | Temperate oceanic | 16.4 | 149.3 |
| 2017^1^ | 50.558596N, 4.716483E | 166 | Silty | Rapeseed | Temperate oceanic | 17.8 | 56.5 |

^1^ : The trial focusing on the 10 contrasted spelt accessions to study interannual variations in the epitope expression and the trial investigating the influence of the N fertilization strategy have been carried out at the same location.

^2^ : Data collected in June (grain filling period) by the unit of agrarian systems, territories and information technology from the Walloon agricultural Research Center (Gembloux, Belgium).
